# DRDarkNet: a hybrid deep feature engineering model for accurate autopsy image classification

**DOI:** 10.1007/s00414-026-03763-8

**Published:** 2026-03-20

**Authors:** Kubra Yildirim, Prabal Datta Barua, Abdurrahim Turkoglu, Nazif Harun Vicdanli, Sengul Dogan, Turker Tuncer, Ilyas Somunkiran, Subrata Chakraborty, Burak Taşcı, Abdul Hafeez Baig

**Affiliations:** 1 Department of Digital Forensics Engineering, College of Technology, Firat University, Elazig, Turkey; 2 School of Business (Information System), University of Southern Queensland, Toowoomba, Australia; 3 Department of Forensic Science, Faculty of Medicine, Firat University, Elazig, Turkey; 4 Department of Forensic Science, Elazig The Council of Forensic Science, Elazig, Turkey; 5 Department of Metallurgical and Materials Engineering, Faculty of Technology, Fırat University, Elazig, 23119 Turkey; 6 School of Science and Technology, Faculty of Science, Agriculture, Business and Law, University of New England, Armidale, NSW 2351 Australia; 7 Center for Advanced Modelling and Geospatial Information Systems, Faculty of Engineering and IT, University of Technology Sydney, Sydney, NSW 2007 Australia; 8 Vocational School of Technical Sciences, Firat University, Elazig, 23119 Turkey; 9 School of Business, University of Southern Queensland, West Street, Toowoomba, QLD Australia

**Keywords:** Autopsy image classification, Pruned iterative majority voting, Deep feature engineering

## Abstract

In deaths due to injury, photographs of changes on deceased bodies are routinely taken during the forensic examination; the task of differentiating the types of fatal injury can be posed as an image classification problem. We aimed to develop a machine learning model for automated classification of the cause of injury-induced deaths based on postmortem images of external body regions. We collected a dataset comprising 4254 autopsy images of various body parts divided into six classes according to the cause of death: (i) crush (1808), (ii) choking (327), (iii) stabbing (977), (iv) gunshot (765), (v) burns (254), and (vi) drowning (127). Our model, DRDarkNet, comprised four phases: feature extraction; feature selection; classification; and information fusion. DenseNet201, ResNet50, and DarkNet53 pre-trained on the ImageNet-1 K dataset were deployed to generate six feature vectors of different lengths using the fully connected and global average pooling layers of the individual networks. Neighborhood component analysis (NCA), Chi2, and ReliefF functions were used to create 18 (= 6 × 3) selected feature vectors of identical length (512) with reduced dimensionality that contained the most discriminative features. These selected feature vectors were then fed to a support vector machine classifier to generate 18 classifier-wise outputs. Novel pruning-based iterative majority voting (PIMV) was used to aggregate the classifier-wise outputs, from which voted outputs were generated. From both classifier-wise and voted outputs, the most accurate output was automatically chosen, rendering the model self-organized. DRDarkNet outputs both classifier-wise results and voted results, attaining an excellent 96.47% overall multiclass classification accuracy.

## Introduction

Forensic experts conduct autopsies to determine the cause of death [1–3]. The findings provide pathoanatomical insights into unexplained deaths and constitute evidence in cases of unnatural deaths [4], which are vital for judicial investigations [5]. During autopsy, external examination and photographic recordings of observed changes on the deceased bodies are routinely performed [6–8]. External bodily changes include blunt injuries, wounds caused by sharp tools, burns, strangulation marks, etc [9, 10]. Analysis of such changes offers crucial clues about the cause of death. However, examining individual changes can be time-consuming, particularly in multiple injuries [1, 11], which may lead to delays in diagnosis [9].

In unnatural deaths due to injury, the forensic task of differentiating the various types of bodily injury leading to death can be posed as an image classification problem to which artificial intelligence methods can be applied. There is a paucity of studies on postmortem external injury classification. Among studies conducted on patients while alive, many have focused on identifying and/or rating wounds [12–17], including burns [18–22], often on limited datasets [12–16]. Fernandes et al. [23] proposed a model that could distinguish between consensual and non-consensual relationships for forensic examination of sexual assaults. ResNet-50 and Inception-v3 classification networks were trained on 394 images of genital injuries (78 and 316 images from non-consensual and consensual relationships, respectively). The model attained a 91% F1 score and 89% C statistic. Alkaissy et al. [24] proposed a machine-learning model for classifying construction site accidental injuries based on the body region location. On a 16,878-image workplace accident dataset stratified by body regions (41.5% upper limbs, 26.5% lower limbs, 9.4% head and neck, and 22.6% back and trunk), re-sampling techniques were applied to balance the classes. The model attained a 78.5% F1 score using a decision tree. Among forensic studies, many were based on postmortem medical imaging data rather than external body images. Garland et al. [25] investigated fatal head injury assessment using postmortem radiological imaging, specifically postmortem transverse computed tomography (PMCT) slices acquired at the frontal sinus level. Their dataset included 25 fatal traumatic brain injury cases and 25 non–head-injury cases, and they reported classification accuracies ranging from 70% to 92.5%. Importantly, this line of work should be interpreted as a postmortem CT–based imaging problem (i.e., radiological slice analysis), which is methodologically different from autopsy image classification as addressed in our study. In our setting, “autopsy images” refer to external gross photographs captured during medico-legal autopsy examinations (surface/body-region images), not radiological CT volumes or axial slices; therefore, the visual evidence, noise sources, and discriminative cues differ substantially between PMCT studies and photographic autopsy-image classification. Malviya et al. [26] discuss the potential of AI to improve precision, reliability, and efficiency in forensic pathology and autopsy analysis. Based on a literature review, they highlight technologies such as VIRTOPSY and artificial neural networks that complement traditional autopsies. The study notes that while AI offers significant benefits, its adoption is shaped by ethical, legal, and technical constraints, ultimately positioning it as a powerful tool in modern forensic practice. Homma et al. [27] investigated drowning cases using lung computed tomography. They studied 3784 and 3863 from 140 drowning and non-drowning cases, respectively, all of whom had undergone autopsy by experienced forensic pathologists. The drowning and non-drowning cases were divided into 14 and 10 groups, respectively. Among these groups, nine were used for transfer learning using pre-trained AlexNet; the remaining group for testing. The model attained 88% accuracy for binary classification of drowning vs. non-drowning cases.

We aimed to develop a machine learning model for automated classification of the cause of injury-induced deaths based on postmortem images of external body regions, which could assist forensic experts during autopsies. Toward this end, a multi-class postmortem image dataset of various injured body regions was collected to train and test our model.

### Motivation and our model

For our digital forensic model, we used computer vision combined with transfer learning methods. Images from a newly acquired forensic dataset were input to a novel DRDarkNet architecture, which used three pre-trained convolutional neural networks (CNNs) to extract deep features in parallel. To reduce data dimensionality, multiple effective feature selection functions were deployed. The selected feature vectors were fed to a standard shallow classifier to generate prediction results. From these, additional voted results were calculated using a modified form of iterative majority voting [28], and the overall most accurate result was chosen from among all generated results.

### Novelties and contributions

Our model featured a novel DRDarkNet feature extraction model based on pre-trained CNNs and an original information fusion method. Our model was self-organizing: the best result was automatically chosen from among classifier-wise and voted outputs. A large autopsy image dataset was collected to train and test the model. Our DRDarkNet attained excellent 96.47% classification accuracy on the collected dataset, demonstrating its feasibility for automated image-based forensic examination of injury-induced deaths.

## Materials and methods

This section describes the materials and methodological framework adopted in the present study. It outlines the characteristics of the autopsy image dataset, followed by detailed descriptions of the deep feature extraction process, feature selection strategies, classification methodology, and the proposed information fusion approach.

### Dataset

**Fig. 1:**
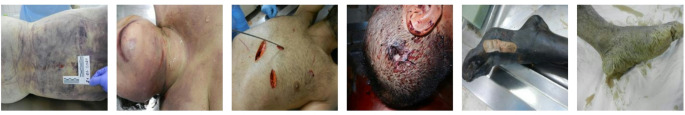
Example dataset images depicting causes of death and body parts involved (from left to right): crushing, back; choking, neck; stabbing, chest; gunshot, head; burns, foot; drowning, foot

The study dataset comprised 4254 RGB autopsy images of various body parts that had been retrospectively collected from 01/01/2015 to 31/12/2021 and stored as.jpg files. Informed consent was not applicable, as this study was based on retrospectively collected medico-legal autopsy images obtained during routine forensic practice. The use of these data was conducted in accordance with national legal regulations governing forensic autopsies and medical research, and all images were fully anonymized prior to analysis. All images were labeled by forensics expert from Firat University, Turkey, and divided into six classes (numbers) according to the cause of death: (i) crushing (1808), (ii) choking (327), (iii) stabbing (977), (iv) gunshot (761), (v) burns (254), and (vi) drowning (127) (Fig. 1). Only cases in which the cause of death was conclusively established through a complete medico-legal autopsy were included in the study. Image selection was restricted to injuries determined to be causally related to death based on the final autopsy conclusions, rather than incidental, superficial, or non-lethal findings. In cases with multiple injuries, images corresponding to the lethal mechanism identified in the official autopsy report were selected. All class labels were assigned and validated by experienced forensic medicine specialists from the Department of Forensic Medicine, Faculty of Medicine, Firat University, and the Elazig Council of Forensic Medicine. Autopsy images were acquired as part of routine forensic documentation under real-world conditions rather than a strictly standardized imaging protocol; therefore, variations in camera type, resolution, illumination, viewing angle, and anatomical field of view may exist. Nevertheless, all photographs were obtained by trained forensic personnel during external examinations and reflect standard medico-legal documentation practices at our institution.

### DRDarkNet

**Fig. 2:**
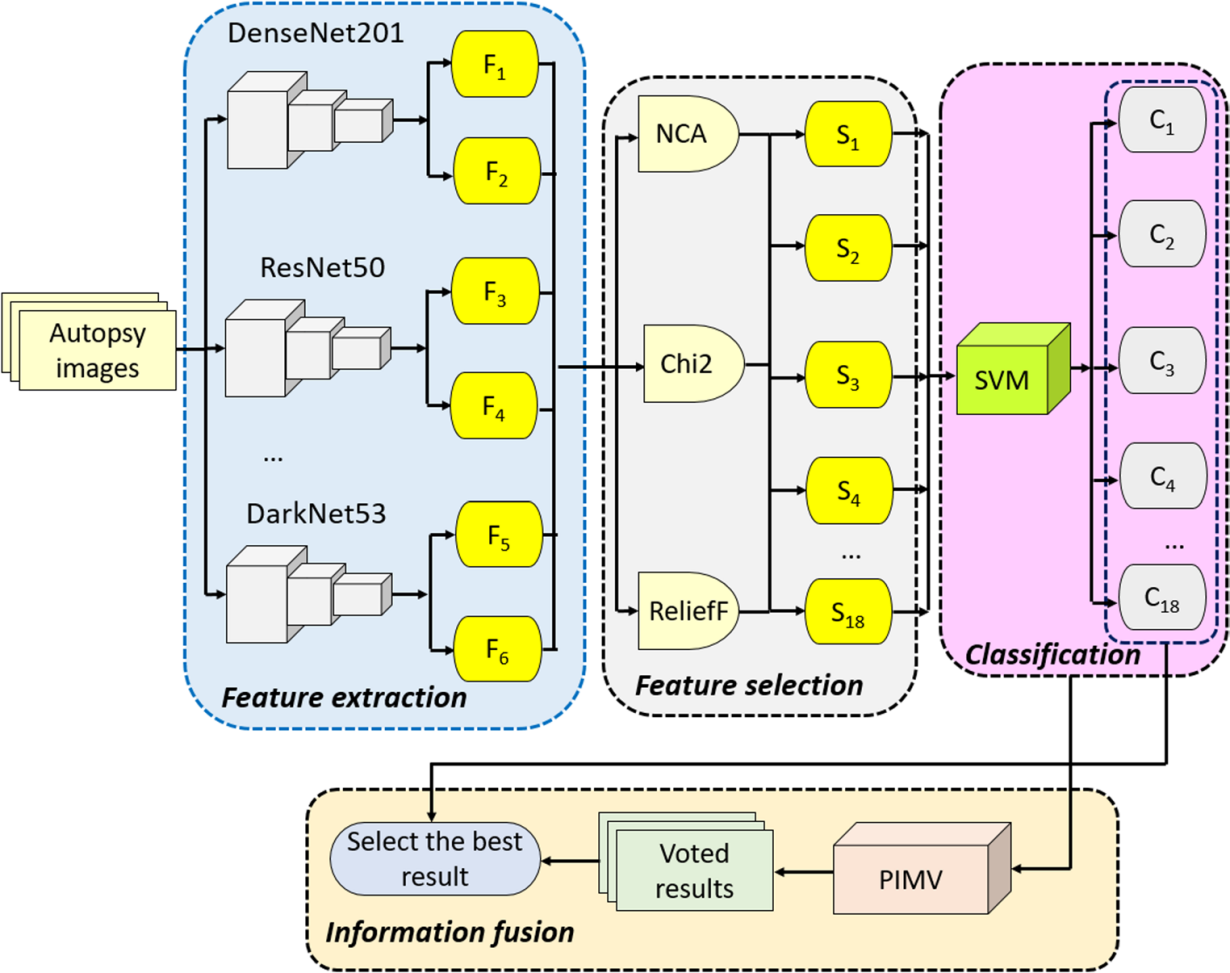
Schema of the proposed model. The DRDarkNet feature-generating architecture

Our model comprised four phases: deep feature extraction; feature selection using three selection functions; classification; and information fusion (Fig. 2).

The architecture consists of DenseNet201 [29], ResNet50 [30], and DarkNet53 [31] pre-trained models trained using ImageNet1k dataset, which generated six feature vectors (Fs) of different lengths using the fully connected and global average pooling layers of the individual networks. Neighborhood component analysis (NCA), Chi2, and ReliefF functions were deployed to create 18 (= 6 × 3) selected feature vectors (Ss) of the same length with reduced dimensionality that contained the most discriminative features, which were then fed to support vector machine (SVM) [32, 33] classifier to generate 18 classifier-wise outputs (Cs). Novel pruning-based iterative majority voting (PIMV) was used to aggregate the classifier-wise outputs, from which voted outputs were generated. From both classifier-wise and voted outputs, the most accurate output was chosen.

### Deep feature extraction

We harnessed transfer learning for deep feature extraction through the deployment of DenseNet201 [29], ResNet50 [30], and DarkNet53 [31], which had been pre-trained on the ImageNet1K dataset. The fully connected and global average pooling layers of each CNN were used to extract deep features, yielding six (= 3 × 2) feature vectors per input image. The steps are detailed below.

- ***Step 1:*** Load the three pre-trained deep networks.
- ***Step 2:*** Generate features using the fully connected layer and global average pooling layers to create six feature vectors.

1 $$\begin{array}{l}{F}_{u}=CN{N}_{k}\left(I,laye{r}_{i}\right),i\in\left\{\mathrm{1,2},3\right\},k\in\left\{\mathrm{1,2}\right\},u\in\\\left\{\mathrm{1,2},\dots,6\right\}CNN\in\left\{DenseNet201,ResNet50,DarkNet53\right\},\\layer=\{FC,GAP\}\end{array}$$

where $FC$ represents the fully connected layer; $GAP$, the global average pooling layer; and $F$, extracted feature vector.

### Feature selection

Three well-known feature selection functions were used. Neighborhood component analysis (NCA) [34] calculates the optimal linear transformation of the features that maximize the accuracy of the nearest neighbor classifier. Focusing on local neighborhood relationships in the data and assessing how effectively each feature discriminates between data points belonging to the same class and those from different classes, NCA identifies features that best contribute to accurate class label predictions. Chi2 [29], a statistical measure, is a fast feature selector for classification tasks involving categorical data. Assuming data independence, the Chi2 statistic is computed from the observed and expected frequency distributions of the feature: higher Chi2 statistic indicates a stronger dependency between the feature and the target variable, signifying feature relevance and discriminative utility for class prediction. ReliefF [35], a distance-based function, quantifies feature relevance from differences in feature values among nearest neighbors to distinguish between data points of the same class and those from different classes. Feature weights are calculated based on these differences: higher weights denote higher discriminative utility of the feature for the classification task. ReliefF can handle mixed-type datasets (including categorical and continuous feature spaces) and is computationally efficient, making it a popular choice for data mining and machine learning applications.

In our model, from the six extracted feature vectors of different lengths, the three feature selectors generated 18 (= 6 × 3) selected feature vectors of identical lengths (512 features) that each contained the most informative features as determined by the respective selection functions. The steps are detailed below.

- ***Step 3:*** Calculate qualified/sorted indexes of the six extracted feature vectors using NCA, Chi2 and ReliefF functions.

2 $$\begin{array}{l}in{d}_{r}^{g}=FeatSe{l}^{g}\left({F}_{r},y\right),u\in\left\{\mathrm{1,2},\dots,6\right\},\\g\in\left\{\mathrm{1,2},3\right\},FeatSel\in\{NCA,Chi2,ReliefF\}\end{array}$$

where $ind$ represents the qualified/sorted indexes; $FeatSel(.)$, feature selection function; and $y$, actual output.

- ***Step 4:*** Select the most informative 512 features using the calculated indexes to generate 18 selected feature vectors.

3 $$\begin{array}{l}{S}_{q}\left(dim,x\right)={F}_{u}\left(dim,in{d}_{u}^{g}\left(x\right)\right),q\in\left\{\mathrm{1,2},\dots,18\right\},\\dim\in\left\{\mathrm{1,2},\dots,NoIm\right\},x\in\{\mathrm{1,2},\dots,512\}\end{array}$$

where $S$ represents the selected feature vector of length 512; and $NoIm$, the number of features.

### Classification

The selected feature vectors were input to a standard shallow classifier, cubic SVM [32, 33], to generate 18 classifier-wise results using a robust 10-fold cross-validation strategy. The steps are detailed below.

- ***Step 5:*** Classify the selected features to generate classifier-wise outputs.

4 $${C}_{q}=SVM\left({S}_{q},y\right)$$

where $C$ represents classifier-wise output; and $SVM\left(.\right),$cubic SVM classifier.

### Information fusion

We introduced a novel modification of the iterative majority voting [28], pruning iterative majority voting (PIMV), which was able to generated additional voted outputs from the aggregated classifier-wise outputs while effectively excluding negatively affected classifier-wise outputs. From both classifier-wise and voted outputs, the output with maximum accuracy was chosen. The pseudocode (Algorithm 1) and detailed steps are provided below.

**Figure:**
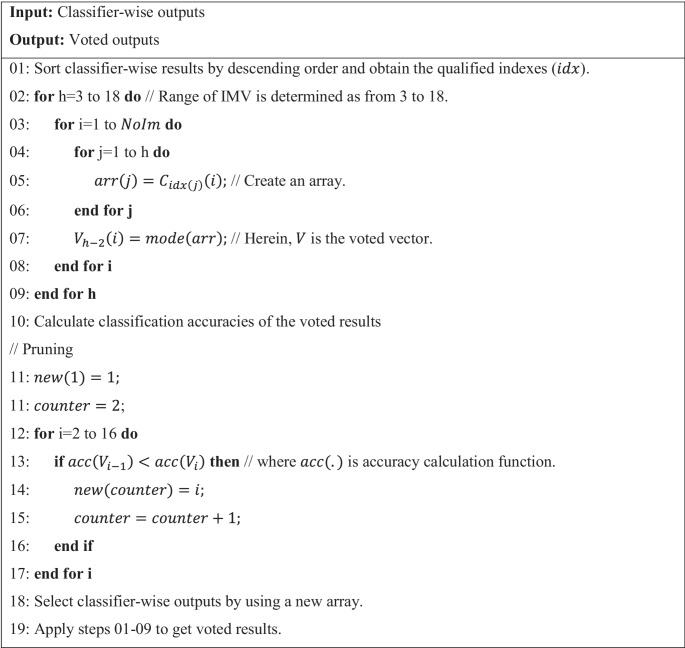
Algorithm 1. Pseudocode for pruning iterative majority voting (which was implemented as a MATLAB .m file)

- ***Step 6:*** Apply PIMV to classifier-wise outputs to generate the voted outputs.

- ***Step 7:*** Compute classification accuracies of all outputs.

5 $$V=PIMV\left(C\right)$$

Where $V$ represents voted output.

6 $${a}_{t}=acc\left(C\right),t\in\left\{\mathrm{1,2},\dots,18\right\}{a}_{r+18}=acc\left({V}_{r}\right),r\in\{\mathrm{1,2},\dots,nv\}$$

where $a$ represents classification accuracy; and $nv$, the number of voted outputs.

- ***Step 8:*** Select the final result based on classification accuracies.

7 $$inx=max\left(a\right)$$

8 $$FinRes=\left\{\begin{array}{c}{C}_{inx},inx\le18\\{V}_{inx-18},inx>18\end{array}\right.$$

where $inx$ represents the index of the results with maximum accuracy; $inx$, the index of the maximum accuracy by deploying the maximum ($max(.)$) function; and $FinRes$, final result.

Steps 1 to 8 above defined our proposed DRDarkNet model.

## Experimental results

### Setup

The images were resized to 224 × 224 before input into the model. The model was implemented in MATLAB programming environment on a personal computer with a 3.6 GHz central processing unit (without the need for the graphics card), 32 GB memory, and Windows 11 operating system. The parameters of the DRDarkNet model are summarized in Table 1.

**Table 1:** Transition table of the DRDarkNet model

Phase	Operation	Parameters	Output
Deep feature extraction	Pre-trained ResNet50, DenseNet201, DarkNet53	Feature extraction layers: FC, GAP	F1: ResNet50 + FC F2: ResNet50 + GAP F3: DenseNet201 + FC F4: DenseNet201 + GAP F5: DarkNet53 + FC F6: DarkNet53 + GAP
Feature selection	NCA, Chi2, ReliefF	Selected feature vector length: 512	S1: ResNet50 + FC + NCA S2: ResNet50 + FC+Chi2 S3: ResNet50 + FC+ReliefF S4: ResNet50 + GAP + NCA S5: ResNet50 + GAP+Chi2 S6: ResNet50 + GAP+ReliefF S7: DenseNet201 + FC + NCA S8: DenseNet201 + FC+Chi2 S9: DenseNet201 + FC +ReliefF S10: DenseNet201 + GAP + NCA S11: DenseNet201 + GAP+Chi2 S12: DenseNet201 + GAP+ReliefF S13: DarkNet53 + FC + NCA S14: DarkNet53 + FC+Chi2 S15: DarkNet53 + FC+ReliefF S16: DarkNet53 + GAP + NCA S17: DarkNet53 + GAP+Chi2 S18: DarkNet53 + GAP+ReliefF
Classification	Cubic SVM	Kernel: 3rd polynomial; C/box constraint value: 1; coding: one-vs-all; validation: 10-fold cross-validation	18 classifier-wise results
Information fusion	PIMV	Range: 3 to 18; function: mode	10 voted results
	Best result selection	Maximum classification accuracy	Best of 28 (= 18 + 10) results

** *F* feature vector, *FC* fully connected, *GAP* global average pooling, *NCA* neighborhood component analysis, *PIMV* pruning iterative majority voting, *S* selected feature vector, *SVM* support vector machine

### Performance evaluation parameters

The model was evaluated using standard performance metrics: accuracy, precision, recall, and F1-score. We have used these performance evaluation metrics to get comprehensive results.

### Results

#### Classifier-wise results

The best classifier-wise accuracy of 95.77% accuracy was attained with the 10th selected feature vector, which was formed using the combination of DenseNet201, global average pooling layer, and NCA (Table 2).

**Table 2:** Classification performance of classifier-wise results calculated by support vector machine

No.	Acc (%)	UAP (%)	UAR (%)	F1 (%)	No.	Acc (%)	UAP (%)	UAR (%)	F1 (%)
1	93.58	92.59	90.20	91.38	10	**95.77**	**95.26**	**93.37**	**94.30**
2	93.61	92.56	90.22	91.38	11	95.39	94.35	93.26	93.80
3	93.42	92.68	89.72	91.18	12	95.42	94.91	93.33	94.11
4	94.22	93.08	90.92	91.99	13	92.81	91.71	88.37	90
5	94.12	92.68	90.68	91.67	14	92.64	90.91	88.03	89.44
6	93.56	92.94	89.61	91.24	15	92.97	91.95	88.70	90.30
7	93.37	92.22	89.48	90.83	16	93.46	92.50	89.32	90.89
8	93.07	90.83	88.87	89.84	17	93.54	92.73	89.36	91.01
9	93.18	92.37	89.29	90.81	18	93.28	92.48	88.81	90.60

** *Acc* Accuracy, *UAP* unweighted average precision, *UAR* unweighted average recall, *F1* F1-score

#### Voted results

Applying PIMV (Algorithm 1) to the 18 classifier-wise results, 10 voted results were obtained after pruning 6 negatively affected classifier-wise outputs. The best-voted accuracy was 96.47% (Table 3), which improved upon the best classifier-wise results by 0.7%.

**Table 3:** Classification performance of voted results generated by pruning iterative majority voting

No.	Acc (%)	UAP (%)	UAR (%)	F1 (%)	No.	Acc (%)	UAP (%)	UAR (%)	F1 (%)
1	95.79	95.26	93.56	94.40	6	96.29	95.93	93.60	94.75
2	96	95.62	93.40	94.50	7	**96.47**	96.02	94.16	95.08
3	96.40	**96.04**	**94.20**	**95.11**	8	96.40	95.89	93.76	94.81
4	96.05	95.84	93.48	94.65	9	96.36	95.92	93.87	94.89
5	96.43	96	94.16	95.07	10	96.10	95.76	93.61	94.67

#### Overall result of the proposed DRDarkNet

**Fig. 3:**
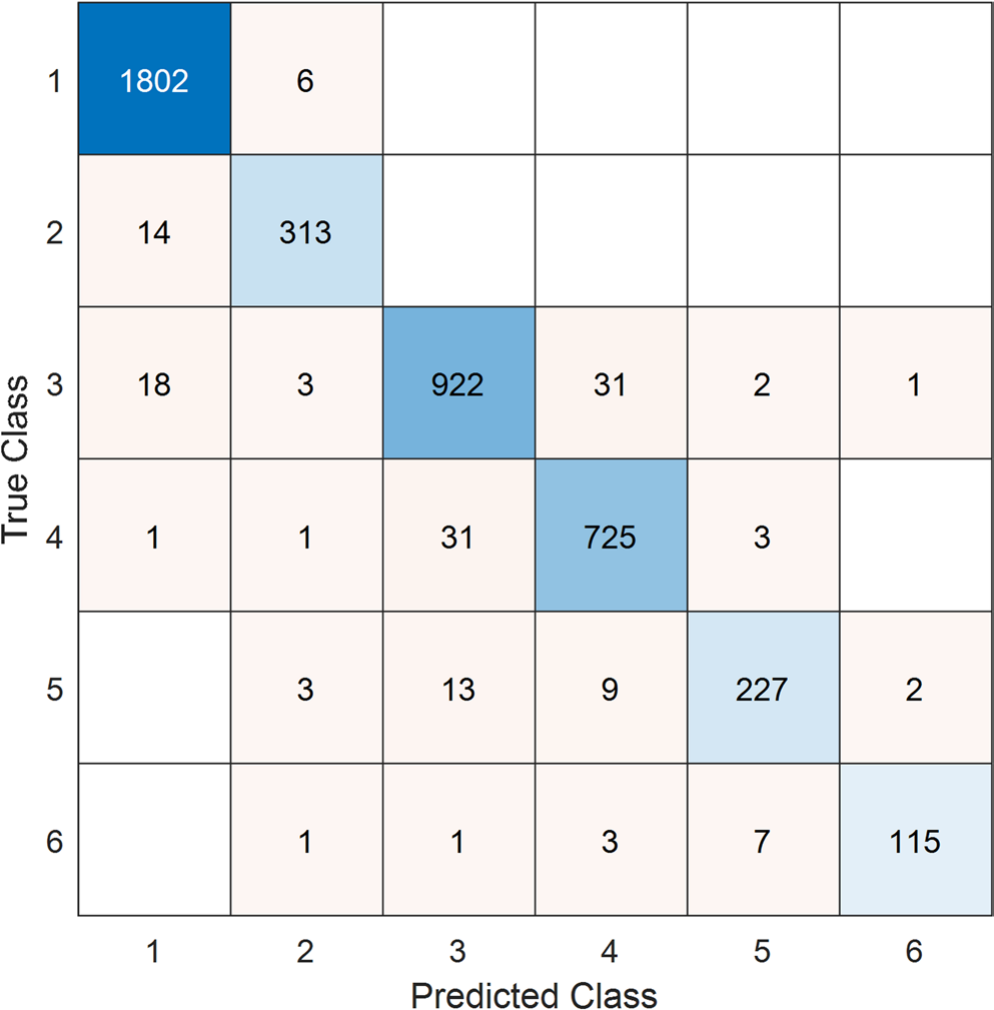
Confusion matrix of the proposed model. Classes 1 to 6 refer to crushing, choking, stabbing, gunshot, burns, and drowning, respectively

Our model automatically selected the best result with the maximum classification accuracy, rendering DRDarkNet a self-organized deep feature engineering architecture. The model attained excellent overall 6-class classification accuracy of 96.47% on the study dataset with low rates of misclassification of the individual classes (Fig. 3), as well as excellent class-wise classification performance, with the best individual class classification performance seen in the crushing class (Table 4).

**Table 4:** Class-wise performance of the DRDarkNet model for autopsy image classification

Class	Accuracy (%)	Precision (%)	Recall (%)	F1-score (%)
Crushing	-	98.20	99.67	98.93
Choking	-	95.72	95.72	95.72
Stabbing	-	95.35	94.37	94.86
Gunshot	-	94.40	95.27	94.83
Burn	-	94.98	89.37	92.08
Drowning	-	97.46	90.55	93.88
Overall	96.47	96.02	94.16	95.08

Analysis of the confusion matrix (Fig. 3) indicates which types of misclassification took place. The majority of misclassifications happened between injury types which shared similar tissue structures or located in adjacent body areas. The external appearance of stabbing wounds might lead to incorrect classification of stabbing cases as gunshot injuries because their external characteristics such as wound shape and size and tissue disruption appear similar. The external examination results from burn and drowning cases contained limited misidentification of cases because the external signs were either hard to detect or lacked clear identification.

The misclassifications do not prove that the system failed to identify visible injuries because they demonstrate how difficult it becomes to identify injury causes through visual inspection of external photographs. The visual appearance of external injuries became less clear because of two factors which included body region depiction and anatomical and contextual information. The process of determining lethal mechanisms in forensic work follows the same method which combines internal examination results with complete case information that goes beyond visual inspection of external evidence.

The visual distinction of sharp force injuries does not explain why the stabbing category shows lower classification performance in forensic settings. Real-world situations show that sharp injuries tend to appear with multiple wounds and combined sharp force mechanisms which create visual heterogeneity and label noise during external image-based classification. The evaluation of wounds becomes challenging because different wound characteristics including size and direction and depth and blood residue patterns create difficulties in identifying distinct tissue structures. The images show different injury patterns from sharp objects because the anatomical region where the injuries occur creates additional confusion. Collectively, these factors may reduce the model’s ability to consistently discriminate sharp force trauma from other injury categories based on external appearance alone.

## Explainable AI (XAI) analysis

To enhance the transparency and interpretability of the proposed DRDarkNet model for forensic practitioners, an explainable artificial intelligence (XAI) analysis was performed using Gradient-weighted Class Activation Mapping (Grad-CAM) [36]. Grad-CAM is a widely used visualization technique for convolutional neural networks that generates class-discriminative localization maps by exploiting the gradients of the predicted class flowing into the final convolutional layers. These maps highlight the image regions that contribute most strongly to the model’s classification decisions. The analysis was applied to the DarkNet53 backbone, which is one of the core feature extraction networks within the proposed DRDarkNet architecture.

**Fig. 4:**
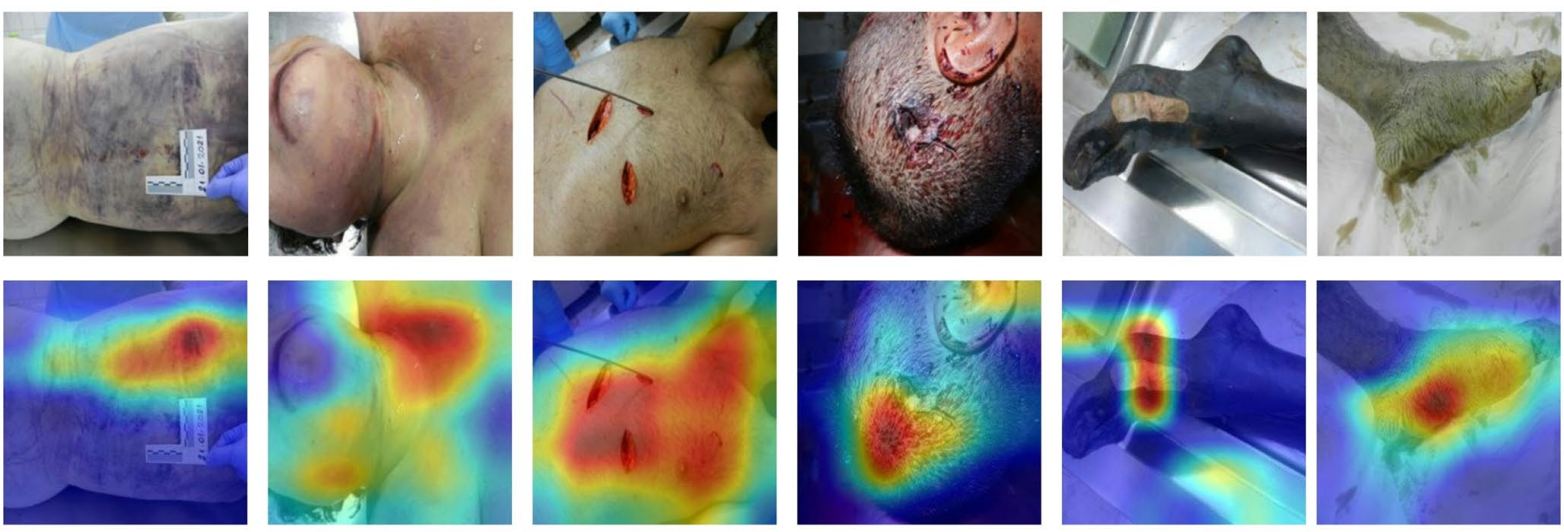
Representative Grad-CAM visualizations for the six cause-of-death categories

Figure 4 presents representative Grad-CAM visualizations for each of the six cause-of-death categories, with the first row showing the original autopsy images and the second row displaying the corresponding Grad-CAM heatmaps overlaid on the images. As illustrated in the figure, the model predominantly focuses on anatomically and morphologically relevant injury regions rather than background or irrelevant image areas. In crushing cases, activation is concentrated over regions exhibiting extensive tissue compression. In choking cases, the highlighted regions are primarily localized around the neck. For stabbing and gunshot injuries, the model attends mainly to wound margins and areas of tissue disruption. Burn-related cases emphasize regions of charring and discoloration, while drowning cases show activation patterns over areas consistent with externally visible postmortem changes.

Overall, the Grad-CAM visualizations provide intuitive visual evidence that the proposed model bases its predictions on forensically meaningful injury characteristics that are consistent with medico-legal reasoning. By explicitly illustrating the image regions influencing classification decisions, this XAI analysis reduces the black-box nature of the model and supports the potential use of DRDarkNet as a decision-support tool in forensic practice. Such explainability mechanisms are particularly important for potential clinical or medico-legal integration, as they support transparency, expert oversight, and compliance with ethical and regulatory expectations in forensic practice.

## Discussion

**Fig. 5:**
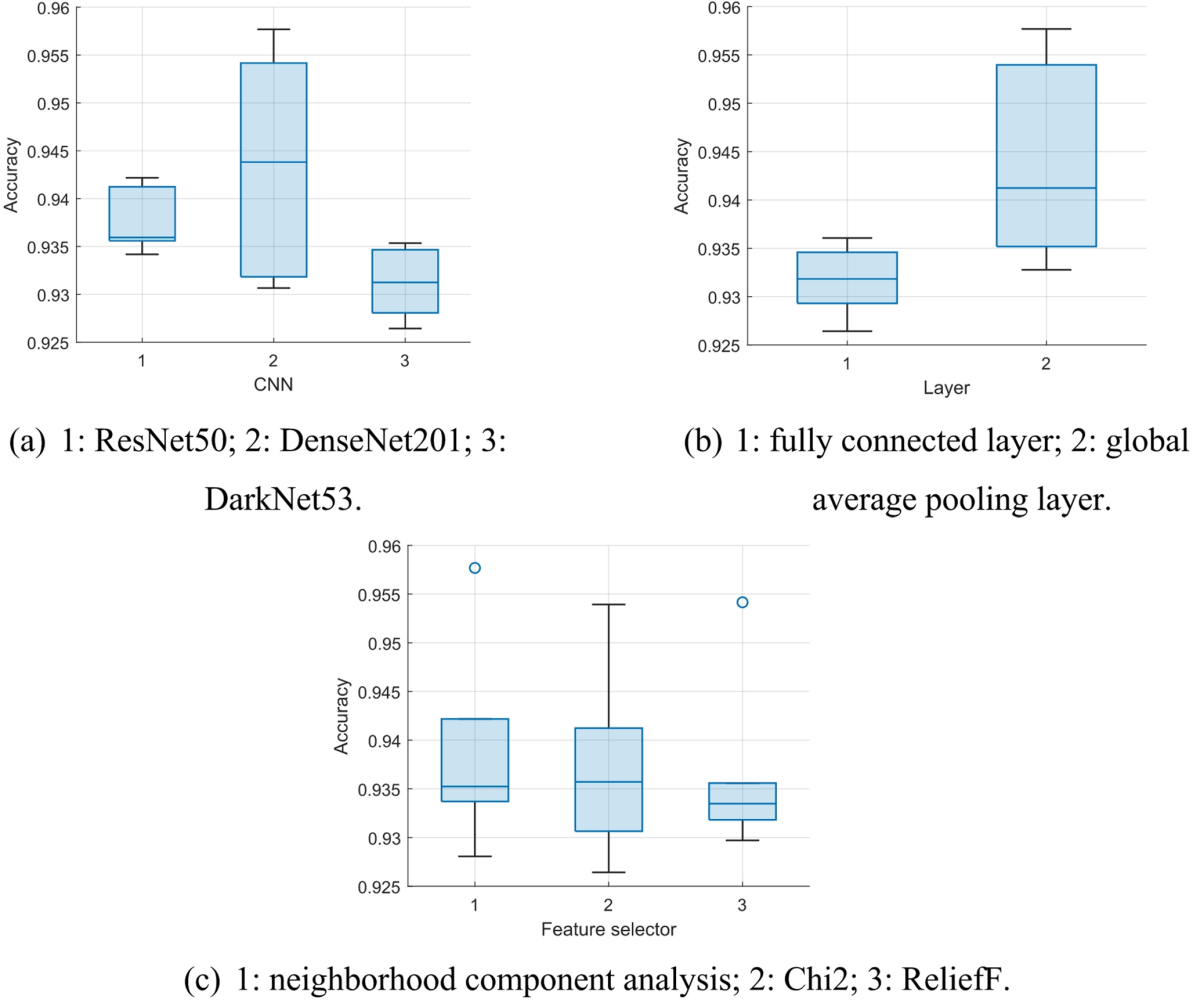
Comparison of the performance of pre-trained networks (**a**), network layers (**b**), and feature selector (**c**) on the study dataset

The DRDarkNet architecture, which comprised three pre-trained CNNs, two network layers, three feature selectors, one classifier, and an information fusion algorithm, generated 18 classifier-wise and 10 voted results, from among which the best overall final result was chosen in a self-organized manner. On the 6-class dataset of autopsy images, the combination of DenseNet201, global average pooling layer, and NCA yielded the highest SVM-calculated accuracy of 95.77%. This combination was corroborated by comparisons of the performance of the various components of the model (Fig. 5), which showed DenseNet201, global average pooling layer, and NCA to be the best performing CNN, network layer, and feature selection function, respectively, in terms of attained overall accuracy.

We acknowledge that contextual and anatomical cues, such as the body region shown (e.g., neck, head, or extremities) or background elements, may have contributed to the classification performance in addition to the visual characteristics of the injury itself. This reflects real forensic practice, where injury patterns are interpreted in conjunction with anatomical localization. Nevertheless, the potential influence of such contextual information represents a limitation of the current study and may introduce bias if images disproportionately emphasize injury-prone regions associated with specific causes of death. Future studies will address this issue by incorporating stricter image standardization, region-of-interest isolation, and multi-center datasets to reduce context-driven bias and improve generalizability.

In real-world practice, DRDarkNet is a decision-support tool for forensic medicine specialists. It is not a standalone system for determining the cause of death. Its role is to support autopsy image documentation and to support consistent interpretation across cases. The explainable artificial intelligence module provides transparency by indicating the image regions associated with the final decision.

Use by non-specialist personnel without full medico-legal context is not appropriate. In forensic medicine, cause-of-death decisions require a complete evaluation beyond external images. In settings with limited forensic resources, DRDarkNet may have value for preliminary triage or educational use, but only with cautious interpretation and expert supervision.

### Ablations

**Fig. 6:**
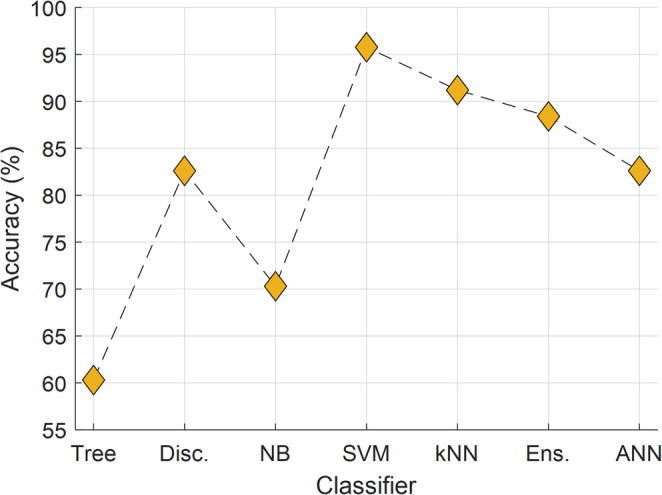
Accuracies obtained with various classifiers on the selected feature vector obtained using the DenseNet201, global average pooling layer, and NCA combination

On the SVM classifier, the combination of DenseNet201, global average pooling layer, and NCA yielded the selected feature vector that produced the best result. This combination was used in the preliminary testing of various standard classifiers in the MATLAB Classification Learner Toolbox. SVM outperformed the other classes of classifiers for testing all datasets by deploying ten-fold cross-validation (Fig. 6), which informed our choice of SVM as the classifier for the DRDarkNet model. Further, we compared the performance of PIMV against iterative majority voting. The former attained 96.47% accuracy employing only nine classifier-wise outputs, which marginally surpassed the latter, which attained 96.45% accuracy using 17 of the 18 available classifier-wise outputs (Fig. 6).

The MATLAB Classification Learner Toolbox contained approximately 30 classifiers, which could be broadly classified into seven categories: tree-based (Tree); discriminant (Disc.); naïve Bayes (NB); support vector machine (SVM), (5) k-nearest neighbors (kNN); ensemble (Ens.); and artificial neural network (ANN).

**Fig. 7:**
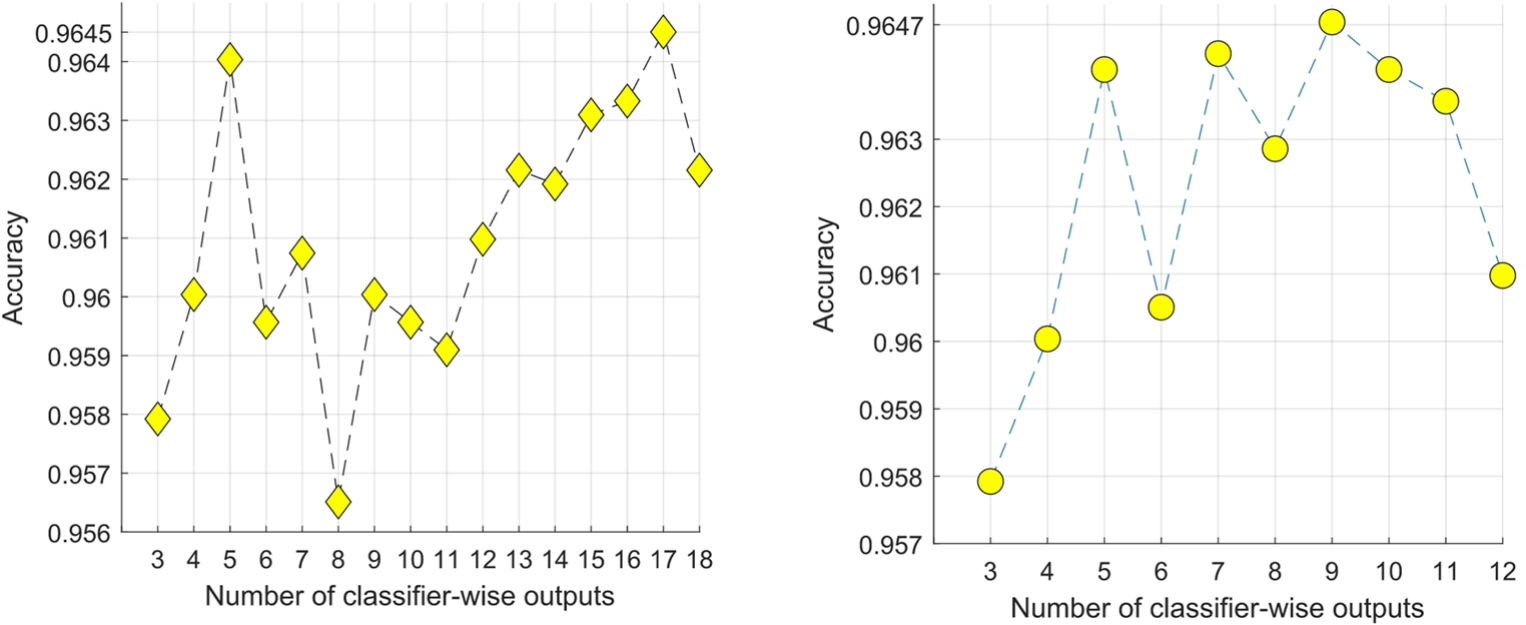
clearly demonstrates that the best classifier among the tested classifiers is SVM

Figure 7. Comparisons of the IMV and PIMV methods. Accuracy of voted results obtained by applying iterative majority voting (left, 16 voted results) and pruning iterative majority voting (right, 10 voted results) on all 18 SVM-calculated classifier-wise results.

We extended the ablation results and we compared feature sizes and these ablation results are tabulated Table 5.

**Table 5:** Ablation results

Configuration	Selected feature	Accuracy (%)
DenseNet201	128	93.98
DenseNet201	256	94.55
DenseNet201	512	95.77
DenseNet201	768	95.39
ResNet50	512	94.22
DarkNet53	512	93.54
DenseNet201 + ResNet50	512	96.12
DRDarkNet	512	96.47

Table 5 presents an extended ablation analysis. It reports the effects of feature size, backbone choice, and fusion on classification accuracy.

First, the feature size analysis is performed with DenseNet201. Accuracy increases from 93.98% at 128 features to 94.55% at 256 features. The best result is obtained with 512 features (95.77%). When the feature size is increased to 768, accuracy slightly decreases to 95.39%. These results indicate that 512 selected features are sufficient for this dataset and that larger feature vectors add limited benefit.

Second, the single-backbone results are compared at 512 features. DenseNet201 gives the highest accuracy (95.77%). ResNet50 reaches 94.22%, and DarkNet53 reaches 93.54%. This ranking indicates that DenseNet201 produces more informative features for external autopsy photographs in this study.

Third, the fusion results are reported. The two-backbone setting (DenseNet201 + ResNet50) reaches 96.12%. The full DRDarkNet configuration reaches 96.47%. These results support the use of multi-backbone fusion and indicate that combining backbones improves overall accuracy.

Finally, an additional test is performed without the FC layer. In this case, the DRDarkNet accuracy decreases by about 0.8% points. This result indicates that FC-based features contribute useful information and improve the final classification.

Table 5 highlights that feature size matters, 512 features are the best option, DenseNet201 is the best single backbone, and fusion improves accuracy.

### Comparative results

**Fig. 8:**
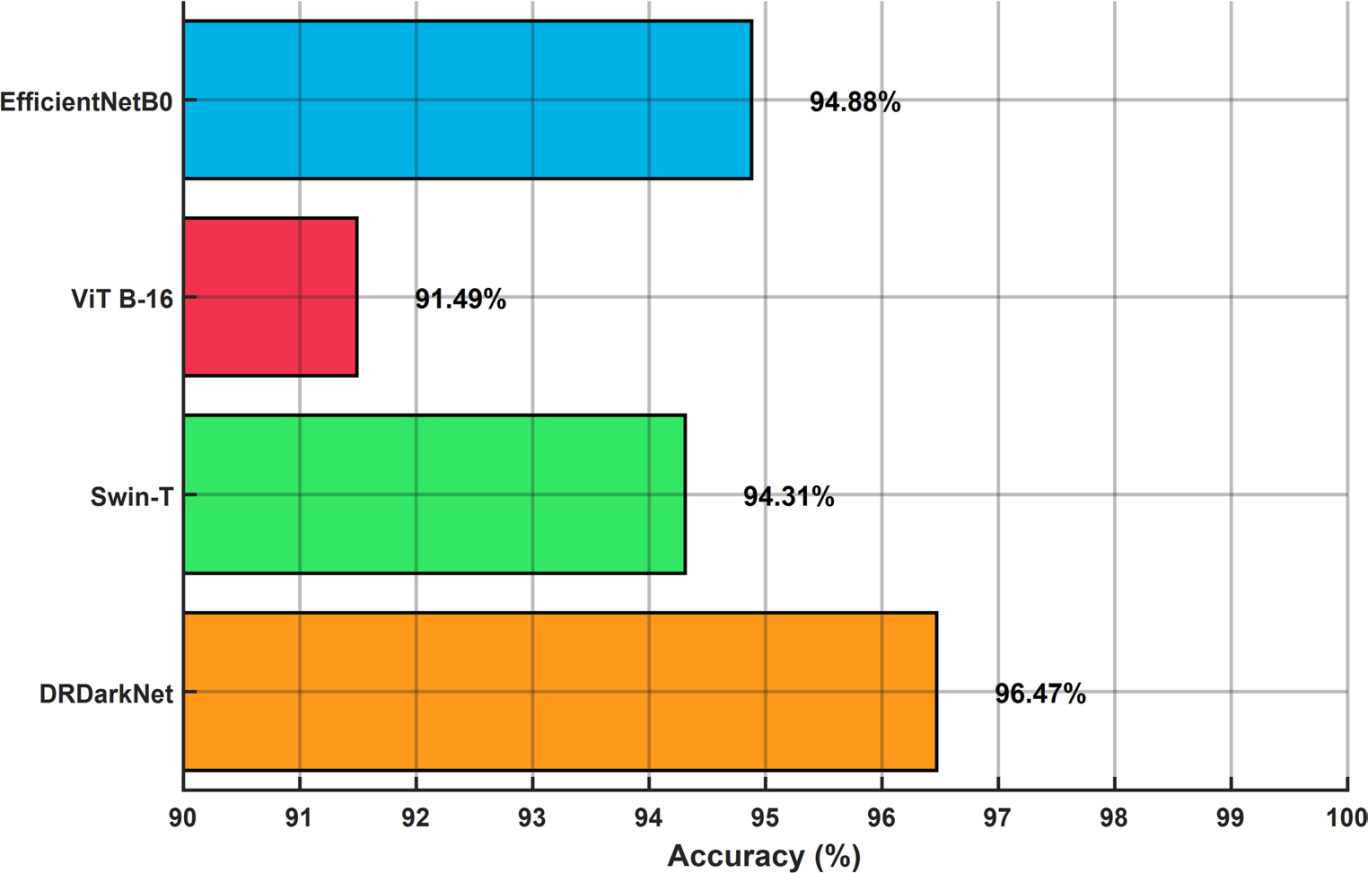
Comparative results

In this study, we propose DRDarkNet, a deep feature engineering framework designed to improve the classification performance of three CNN backbones. To evaluate its effectiveness, we compare DRDarkNet with representative state-of-the-art models. Since the dataset size is relatively small, fully end-to-end training may not produce stable or optimal results. Therefore, we adopt a deep feature extraction strategy to provide a fair comparison. For this purpose, EfficientNetB0, ViT B-16, and Swin-T are used as baseline models, and their classification accuracies are reported in Fig. 8.

Figure 8 compares the classification accuracy of DRDarkNet with three common state-of-the-art baselines (EfficientNetB0 [37], ViT-B/16 [38], and Swin-T [39]) under the same evaluation setting. The results are clear: DRDarkNet achieves the highest accuracy (96.47%), while EfficientNetB0 reaches 94.88%, Swin-T reaches 94.32%, and ViT B-16 reaches 91.49%. In absolute terms, DRDarkNet increases accuracy by + 1.59% over EfficientNetB0, + 2.16% over Swin-T, and + 4.98% over ViT B-16. This consistent improvement indicates that DRDarkNet provides a more reliable decision output for multiclass forensic image classification.

The baseline ranking is also meaningful. EfficientNetB0 is a strong CNN baseline and performs well on medium-sized datasets because it captures texture and local patterns effectively. Swin-T is a modern transformer model and performs close to EfficientNetB0, but it does not surpass DRDarkNet in this experiment. ViT B-16 shows the lowest accuracy, which is expected because standard ViTs usually need larger datasets and stronger training strategies to reach CNN-level performance in domain-specific image tasks.

Figure 8 highlights the main contribution of the proposed method. DRDarkNet benefits from combining multiple deep feature extractors, reducing noise with feature selection, and improving stability with decision fusion. This hybrid structure is important for forensic images where visual evidence can be complex and variable. Therefore, the results in Fig. 8 demonstrate that DRDarkNet is competitive with strong CNN and transformer baselines and achieves the best accuracy in this comparison.

### Highlights

We trained and tested the DRDarkNet model on a new autopsy image dataset, which to our knowledge, was the most extensive. Our model attained excellent overall 6-class and as well individual class classification performance, which attested to the ability of the pre-trained CNNs to extract relevant deep features from the forensic images. Among the model components, DenseNet201, global average pooling layer, and NCA were demonstrated to be the best performing CNN, network layer, and feature selection function, respectively, in terms of overall accuracy. For individual classes, the best classification performance was observed for the crush class. The addition of novel PIMV-based information fusion conferred incremental accuracy using fewer classifier-wise outputs. Further, by incorporating an algorithm that selected the best overall result, the model became fully self-organized, and therefore more suited for automated diagnostic applications. Through the use of transfer learning, training times were reduced compared with training deep models from scratch. Overall, the components of our model were computationally lightweight, which enhanced the ease of implementation.

## Limitations and future works

The presented DRDarkNet is a self-organized deep feature engineering model. In this aspect, it contributes to both feature engineering and deep learning, but it has a few limitations.

First, although the dataset is relatively large, it was collected from a single medical center. This limits cross-center generalizability. In real practice, imaging conditions differ across institutions due to camera type, sensor quality, lens properties, resolution, illumination, shooting distance, angle, background, and documentation routines. These differences may cause domain shift and reduce performance on external datasets. Future work will include multi-center data collection, external validation, and controlled robustness tests under systematic variations such as illumination change, blur, compression, scale, rotation, and partial occlusion. We will also study domain adaptation and standardization strategies, including color normalization, exposure correction, and test-time adaptation, to improve transfer across centers.

Second, the current study uses pre-trained CNNs mainly as fixed feature extractors. Fine-tuning may improve performance, but it can increase training cost and overfitting risk. In future work, we will evaluate several training strategies, including partial fine-tuning, progressive unfreezing, and lightweight adaptation methods. We will report accuracy together with compute cost and training time to provide a clear complexity–performance profile. In addition, we will expand comparative experiments with new-generation models, including recent CNN families and transformer-based architectures, under the same evaluation protocol and with macro-level metrics for imbalanced data.

Third, the feature selection stage uses a limited set of selectors and mainly fixed feature lengths. Future work will expand ablations to include wider feature lengths, stability analysis across folds, and iterative feature selection strategies [40]. We will also analyze the sensitivity of the pipeline to the selected feature size and decision thresholds, and we will report confidence intervals to improve statistical reliability. Moreover, we will study alternative fusion strategies and reliability-weighted voting to strengthen the self-organized decision mechanism.

Fourth, we will develop new deep learning models that perform feature engineering inside the network. In this direction, feature selection, feature fusion, and decision fusion will be implemented as trainable, self-organized modules. The goal is to build new-generation deep feature engineering networks that maintain high accuracy on small forensic datasets by enforcing structured representation learning, redundancy control, and stability constraints. We will also design compact versions of these models for low-resource environments, and we will investigate knowledge distillation so that a smaller model can retain the accuracy of the full pipeline.

Fifth, we will extend the method to address practical forensic needs. A single external image may not capture the full context of a case. Therefore, future work will include case-level modeling with multi-view photographs, and we will integrate structured autopsy findings and metadata when available. We will also evaluate open-set behavior and unknown-class detection, since real-world cases may include injury types outside the training set. In addition, we will include calibration and uncertainty estimation so that the system can flag low-confidence decisions and support safer use.

Finally, we will improve interpretability and reproducibility. Future work will provide a systematic error analysis, class-wise and center-wise performance reporting, and a clear data split policy to avoid leakage. We will also release standardized evaluation scripts and benchmark protocols to support fair comparison and future research. Overall, these future studies aim to produce a robust and generalizable decision-support framework and to introduce new deep feature engineering deep learning models that achieve high performance on small datasets with controlled complexity and improved reliability.

## Conclusions

We have developed DRDarkNet, a novel self-organized and computationally lightweight automated autopsy image classification model that encompassed pre-trained DenseNet201, ResNet50, and DarkNet53 for deep feature extraction as well as downstream handcrafted feature engineering components, including a novel PIMV-based information fusion method. The attained excellent 96.47% multiclass classification accuracy on a large forensic image dataset provided indirect support for the ability of the pre-trained CNNs to extract important features from the input images. Further, the PIMV was able to enhance model classification accuracy using fewer classifier-wise outputs. DRDarkNet represents a significant advancement in autopsy image classification; the promising results underline its potential for machine learning-based automation in this domain.

## Data Availability

The data presented in this study are available on request from the corresponding author. The data are not publicly available due to restrictions regarding the Ethical Committee Institution.

## References

[1] Campobasso CP, Introna F (2001) The forensic entomologist in the context of the forensic pathologist’s role. Forensic Sci Int 120:132–139 11457621 10.1016/s0379-0738(01)00425-x

[2] Zerlauth J-B, Doenz F, Dominguez A et al (2013) Surgical interventions with fatal outcome: utility of multi-phase postmortem CT angiography. Forensic Sci Int 225:32–41 22721937 10.1016/j.forsciint.2012.05.013

[3] Likkachai K, Wongwaisayawan S, Siriwes K, Worasuwannarak W (2025) Abbreviated injury scale-guided assessment of traumatic deaths: postmortem CT versus autopsy. Forensic Sci Int Synergy 10:100588 40395462 10.1016/j.fsisyn.2025.100588 PMC12090232

[4] Yeow WL, Mahmud R, Raj RG (2014) An application of case-based reasoning with machine learning for forensic autopsy. Expert Syst Appl 41:3497–3505

[5] Leth PM (2007) The use of CT scanning in forensic autopsy. Forensic Sci Med Pathol 3:65–9 25868893 10.1385/FSMP:3:1:65

[6] Schuliar Y, Knudsen PJT (2012) Role of forensic pathologists in mass disasters. Forensic Sci Med Pathol 8:164–73 22160735 10.1007/s12024-011-9300-3

[7] Skopp G (2004) Preanalytic aspects in postmortem toxicology. Forensic Sci Int 142:75–100 15172073 10.1016/j.forsciint.2004.02.012

[8] Jacques R, Siydock L (2024) Medicolegal Death Investigations and the Autopsy. Forensic Pathology: Death Investigation Bioethics and Other Medicolegal Principles. Springer. pp. 3–51

[9] James SH, Nordby JJ (2002) Forensic science: an introduction to scientific and investigative techniques. CRC

[10] Borsay BA, Halasi BD, Porszasz RK, Gergely PA (2025) Homicide-suicide as domestic violence: a case report with a little literature review. Legal Med 72:102557 39637627 10.1016/j.legalmed.2024.102557

[11] Galante N, Franceschetti L, Del Sordo S, Casali MB, Genovese U (2021) Explosion-related deaths: an overview on forensic evaluation and implications. Forensic Sci Med Pathol 17:437–48 34196925 10.1007/s12024-021-00383-z PMC8413147

[12] Anisuzzaman D, Patel Y, Rostami B, Niezgoda J, Gopalakrishnan S, Yu Z (2022) Multi-modal wound classification using wound image and location by deep neural network. Sci Rep 12:20057 36414660 10.1038/s41598-022-21813-0 PMC9681740

[13] Suha SA, Sanam TF (2022) A deep convolutional neural network-based approach for detecting burn severity from skin burn images. Machine Learning with Applications 9:100371

[14] Carrión H, Jafari M, Bagood MD, Yang H-y, Isseroff RR, Gomez M (2022) Automatic wound detection and size estimation using deep learning algorithms. PLoS Comput Biol 18:e1009852 35275923 10.1371/journal.pcbi.1009852 PMC8942216

[15] Chang CW, Lai F, Christian M et al (2021) Deep learning–assisted burn wound diagnosis: diagnostic model development study. JMIR Medical Informatics 9:e22798 34860674 10.2196/22798 PMC8686480

[16] Blanco G, Traina AJ, Traina JC et al (2020) A superpixel-driven deep learning approach for the analysis of dermatological wounds. Comput Methods Programs Biomed 183:105079 31542688 10.1016/j.cmpb.2019.105079

[17] Kalb EB, Tamsen F, Thiblin I (2025) Rhodizonate, histological analysis of gunshot wounds in autopsy cases. Forensic Sci Int Rep. 10.1016/j.fsir.2025.100424

[18] Abubakar A, Ugail H, Bukar AM (2020) Can machine learning be used to discriminate between burns and pressure ulcer? Intelligent Systems and Applications: Proceedings of the 2019 Intelligent Systems Conference (IntelliSys) Volume 2. Springer. pp. 870 – 80

[19] Shenoy VN, Foster E, Aalami L, Majeed B, Aalami O (2018) Deepwound: Automated postoperative wound assessment and surgical site surveillance through convolutional neural networks. 2018 IEEE International Conference on Bioinformatics and Biomedicine (BIBM). IEEE. pp. 1017-21

[20] Rostami B, Anisuzzaman D, Wang C, Gopalakrishnan S, Niezgoda J, Yu Z (2021) Multiclass wound image classification using an ensemble deep CNN-based classifier. Comput Biol Med 134:104536 34126281 10.1016/j.compbiomed.2021.104536

[21] Monroy B, Bacca J, Sanchez K, Arguello H, Castillo S (2021) Two-step deep learning framework for chronic wounds detection and segmentation: A case study in Colombia. 2021 XXIII Symp Image Signal Process Artif Vis (STSIVA) IEEE:1–6

[22] Kankonkar R, Chetan K (2025) Comprehensive study of fatal burn injuries: 10-year retrospective autopsy study in Goa Medical College, Goa. Int J Forensic Sci 10:1–5

[23] Fernandes K, Cardoso JS, Astrup BS (2018) A deep learning approach for the forensic evaluation of sexual assault. Pattern Anal Appl 21:629–640

[24] Alkaissy M, Arashpour M, Golafshani EM et al (2023) Enhancing construction safety: machine learning-based classification of injury types. Saf Sci 162:106102

[25] Garland J, Ondruschka B, Stables S et al (2020) Identifying fatal head injuries on postmortem computed tomography using convolutional neural network/deep learning: a feasibility study. J Forensic Sci 65:2019–22 32639630 10.1111/1556-4029.14502

[26] Malviya R, Jain A, Lal S, Arora MK, Kumar S (2025) Exploring Artificial Intelligence (AI) in Forensic Pathology and Autopsy Analysis. Forensic Intelligence and Deep Learning Solutions in Crime Investigation. 125 – 46

[27] Homma N, Zhang X, Qureshi A et al (2020) A deep learning aided drowning diagnosis for forensic investigations using post-mortem lung CT images. 2020 42nd Annual International Conference of the IEEE Engineering in Medicine & Biology Society (EMBC). IEEE. pp. 1262-5 10.1109/EMBC44109.2020.9175731 33018217

[28] Poyraz E, Cruz RL (2011) Distributed opinion estimation using iterative majority voting. 2011 45th Annual Conference on Information Sciences and Systems. IEEE. pp. 1–6

[29] Huang G, Liu Z, Van Der Maaten L, Weinberger KQ (2017) Densely connected convolutional networks. Proceedings of the IEEE conference on computer vision and pattern recognition. pp. 4700-8

[30] He K, Zhang X, Ren S, Sun J (2016) Deep residual learning for image recognition. Proceedings of the IEEE conference on computer vision and pattern recognition. pp. 770-8

[31] Redmon J, Farhadi A (2017) YOLO9000: better, faster, stronger. Proceedings of the IEEE conference on computer vision and pattern recognition. pp. 7263-71

[32] Vapnik V (1998) The support vector method of function estimation. Nonlinear Modeling. Springer. pp. 55–85

[33] Vapnik V (2013) The nature of statistical learning theory. Springer science & business media

[34] Goldberger J, Hinton GE, Roweis S, Salakhutdinov RR (2004) Neighbourhood components analysis. Adv Neural Inf Process Syst 17:513–20

[35] Kononenko I (1994) Estimating attributes: Analysis and extensions of RELIEF. European conference on machine learning. Springer. pp. 171 – 82

[36] Selvaraju RR, Cogswell M, Das A, Vedantam R, Parikh D, Batra D (2017) Grad-cam: Visual explanations from deep networks via gradient-based localization. Proceedings of the IEEE international conference on computer vision. pp. 618 – 26

[37] Tan M, Le Q (2019) Efficientnet: Rethinking model scaling for convolutional neural networks. International conference on machine learning. PMLR. pp. 6105-14

[38] Dosovitskiy A (2020) An image is worth 16x16 words: Transformers for image recognition at scale. arXiv preprint arXiv:201011929

[39] Liu Z, Lin Y, Cao Y et al (2021) Swin transformer: Hierarchical vision transformer using shifted windows. Proceedings of the IEEE/CVF international conference on computer vision. pp. 10012-22

[40] Tuncer T, Dogan S, Özyurt F, Belhaouari SB, Bensmail H (2020) Novel multi center and threshold ternary pattern based method for disease detection method using voice. IEEE Access 8:84532–84540

